# Supplementary material for: Modified Desolvation Method Enables Simple One-Step Synthesis of Gelatin Nanoparticles from Different Gelatin Types with Any Bloom Values
Source: Pharmaceutics. 2021 Sep 22;13(10):1537. doi: 10.3390/pharmaceutics13101537 (PMC8541285; doi:10.3390/pharmaceutics13101537)
Supplement: Supplementary file 1 [file pharmaceutics-13-01537-s001.zip › pharmaceutics-1338709-SM.pdf]

pharmaceutics

MDPI

Supplementary Materials: Modified Desolvation Method Enables Simple One-Step Synthesis of Gelatin Nanoparticles from Different Gelatin Types with Any Bloom Values

Pavel Khramtsov, Oksana Burdina, Sergey Lazarev, Anastasia Novokshonova, Maria Bochkova, Valeria Timganova, Dmitriy Kiselkov, Artem Minin, Svetlana Zamorina and Mikhail Rayev

Optimization experiments

Preliminary assessment of factors affecting size and yield of gelatin nanoparticles: small-scale syntheses with gelatin B 75 bloom.

In 2 ml centrifuge tubes 200 µL of gelatin solution was added. Tubes were kept in a dry-block thermostat at +37 °C. Ethanol (96%), methanol (99.8%), and isopropyl alcohol (99.8%) were prewarmed in the water bath at +37 °C. Alcohol was added to the gelatin solution, then mixed for 5 min on a rotator mixer (10 rpm, 360 degrees), and kept in the thermostat at +37 °C for 30 min. 45 µL of 0.8% glutaraldehyde solution was added to each tube, mixed, and left in the thermostat as described above. Unreacted glutaraldehyde was quenched by adding 100 µL of 1 M glycine. Tubes were mixed and kept in the thermostat for 60 min (glycine addition was omitted for samples with initial gelatin concentrations of 2% and 4% due to partial nanoparticle aggregation). Cross-linked nanoparticles were centrifuged at 10000 g and washed with water 4 times. After each centrifugation cycle nanoparticles were redispersed by sonication (10 s, 60% amplification, 3 mm probe, approx. 8 W). Purified nanoparticles were stored at +4 °C. The size of nanoparticles was measured immediately after the preparation. The concentration of gelatin was determined when all the samples were synthesized. The storage period of individual bathes varied from 1 week to 2 months. Before protein quantification nanoparticles were sonicated to obtain homogeneous suspension (10 s, 60% amplification, 3 mm probe, approx. 8 W).

In the course of the experiment, we varied the pH of gelatin solution (pH values were 8, 9, and 10), the concentration of gelatin (8, 16, and 32 mg/mL), and the volume of added alcohol (600 and 1000 µL).

Optimization of gelatin nanoparticles preparation: gelatin B, 75 bloom, gelatin B, 225 bloom, fish gelatin, gelatin A, 62 bloom, gelatin A, 180 bloom.

Ethanol (96%), isopropyl alcohol (99.8%), and gelatin solutions were prewarmed in the water bath at +37 °C. In 50 ml centrifuge tubes containing 4 ml of gelatin solution, a certain volume of ethanol or isopropyl alcohol was added. Tubes were briefly and gently mixed on a rotating mixer (360 degrees, 10 rpm, 5 rounds) and kept in a thermostat at +37 °C for 30 min. Nine hundred microliters of 0.8% glutaraldehyde solution were added to each tube, which was gently mixed (as in the previous step), and left in the thermostat for another 30 min. Cross-linked nanoparticles were transferred to 85 ml polycarbonate centrifuge tubes and centrifuged at 15000 g for 60 min. Sediment was redispersed in the 2 mL of deionized water (when the amount of nanoparticles was too high they were redispersed in 4 ml of water) using sonication and pipette-assisted mixing. Concentrated suspension of gelatin nanoparticles was quantitatively transferred into 2 mL centrifuge tubes (1 mL of nanoparticle suspension per tube) and centrifuged two times at 20000 g for 30 min. After each centrifugation cycle, the supernatant was removed, and nanoparticles were redispersed in 900 or 1000 µL of deionized water (volume of water was decreased when pellet was large) by sonication (10 s, 60% amplification, 3 mm probe, approx. 8 W). Purified nanoparticles were stored at +4 °C. The size of nanoparticles was measured immediately after the preparation. The concentration of gelatin was determined when all the samples were synthesized. The storage period of individual bathes varied from 1 week to 3

months. Before protein quantification nanoparticles were sonicated to obtain homogeneous suspension (10 s, 60% amplification, 3 mm probe, approx. 8 W).

In the course of the experiment, we varied the pH of gelatin solution (pH values were 9 and 10), the concentration of gelatin (5, 9, and 18 mg/mL), and the volume of added alcohol (12, 20, and 28 mL). The pH of the gelatin solution was adjusted by 1 M NaOH, which volume was negligible in relation to the volume of gelatin and, therefore, did not affect the final concentration of gelatin.

Determination of nanoparticle yield. Nanoparticles were homogenized by brief sonication and diluted in phosphate buffer, pH 7. Trypsin was added to the final concentration of 10 μg/mL. Samples were incubated at +37 °C in the thermostat until the solution became clear (OD values at 600 nm as low as in nanoparticle-free samples). Gelatin calibrators were treated in the same way. Twenty-five microliters of digested samples were transferred to a 96-well plate; then 200 μL of BCA reagent was added, and the resulting mixture was incubated for 30 min at +37 °C in the plate thermostat (400 rpm). Absorbance was measured at 562 nm.

Size of nanoparticles was determined by the DLS technique. For DLS measurements nanoparticles were diluted at 1:375 in water. Hereinafter z-average hydrodynamic diameters (D_h) are given.

Results of optimization experiments

The first part of optimization experiments

Firstly, we conducted preliminary desolvation experiments only with gelatin B 75 bloom in order to trace the overall relationship between synthesis conditions and nanoparticle characteristics (size, polydispersity, and yield). Due to the large number of samples we minimized the starting volume of the gelatin solution to 200 μL. One of three types of alcohol (methanol, ethanol, or isopropyl alcohol) was added to the gelatin solution, then nanoparticles were cross-linked and washed. We varied pH (from 8 to 10) and concentration (from 8 to 32 mg/mL) of gelatin solution as well as the volume of alcohol (600 and 1000 μL, which gave gelatin to alcohol ratios of 1:3 and 1:5, respectively). Here and in the following experiments, the number of glutaraldehyde molecules was at least in two-fold excess to the number of lysine residues, providing a sufficient degree of cross-linking [85]. Excess of glutaraldehyde has no significant effect on particle size [86,87]. The addition of diluted glutaraldehyde allowed a decrease in its local concentration and prevented the aggregation of gelatin molecules. Syntheses were made in 2 ml centrifuge tubes, however, the small size of tubes led to imperfect mixing conditions that could affect both the size and yield of nanoparticles. That is why we considered this experiment only as a preliminary study. During the second iteration of experiments, we used larger volumes of reagents (see below in this section) and obtained more consistent results.

The size and yield of nanoparticles decreased with pH increasing when methanol and ethanol were used as non-solvents (Figures S1 and S2). Gelatin molecules have a more negative charge at higher pH values which leads to stronger electrostatic repulsion, making them less susceptible to desolvation. Higher volumes of alcohols provided higher yields, which is explained by decreased solubility of protein at a high alcohol concentration [88]. Importantly, the desolvating efficiency of isopropyl alcohol is significantly higher (yields varied from 70 to 100%) in comparison with ethanol and methanol. The addition of isopropyl alcohol to 80% (1000 μL) provided quantitative desolvation of gelatin independent of other experimental conditions. Counterintuitive decrease of yield in samples with the lowest gelatin concentration can be explained by loss of nanoparticles during washing steps. Such a loss was inevitable due to low sample volumes and had the highest impact in samples with a gelatin concentration of 8 mg/ml because of the low total amount of gelatin in these samples. In general, smaller nanoparticles were obtained when ethanol and methanol were utilized.

The percentage of gelatin transformed to nanoparticles decreased with the increase of initial gelatin concentrations (except for samples with a maximum volume of isopropyl alcohol), whereas the size of nanoparticles increased. Most of the samples prepared at

starting gelatin concentration of 32 mg/mLcontained polydisperse microparticles and submicron particles.

The second part of optimization experiments

The addition of methanol and ethanol provided smaller nanoparticles, however, isopropyl alcohol gave higher yields. Therefore in the second part of the experiments, we used ethanol and isopropyl alcohol as desolvating agents. The volume of gelatin solution was increased to 4 mL and synthesis was performed in 50 mL centrifuge tubes to provide better mixing conditions. Higher pH values (9 and 10 for gelatin B; 10 and 11 for gelatin A and fish gelatin) were used to obtain smaller and more homogeneous nanoparticles. Alcohol to gelatin volume ratios were 3:1, 5:1, and 7:1. Gelatin concentrations were 5, 9, and 18 mg/mL. We decided not to use higher gelatin concentrations because aggregation was observed in the preliminary study at a concentration of 32 mg/mL. For some batches of gelatin, nanoparticles yields of more than 100% were obtained. Overestimation probably occurred across all samples and was caused by the interaction of free aldehyde groups located on the nanoparticles' surface with a BCA reagent that was used for gelatin quantification [89]. Nevertheless, the general effect of synthesis conditions on the nanoparticle yield still could be assessed.

Surprisingly, much more consistent results were obtained when optimization was conducted at a larger scale. Even at the highest gelatin concentration, monodisperse nanoparticles were obtained, indicating the possibility of further increase of gelatin concentration. Data on the size and yield of nanoparticles are summarized in the figures S3–S6**.** Only one batch of nanoparticles was prepared for each set of conditions, therefore obtained results are not conclusive.

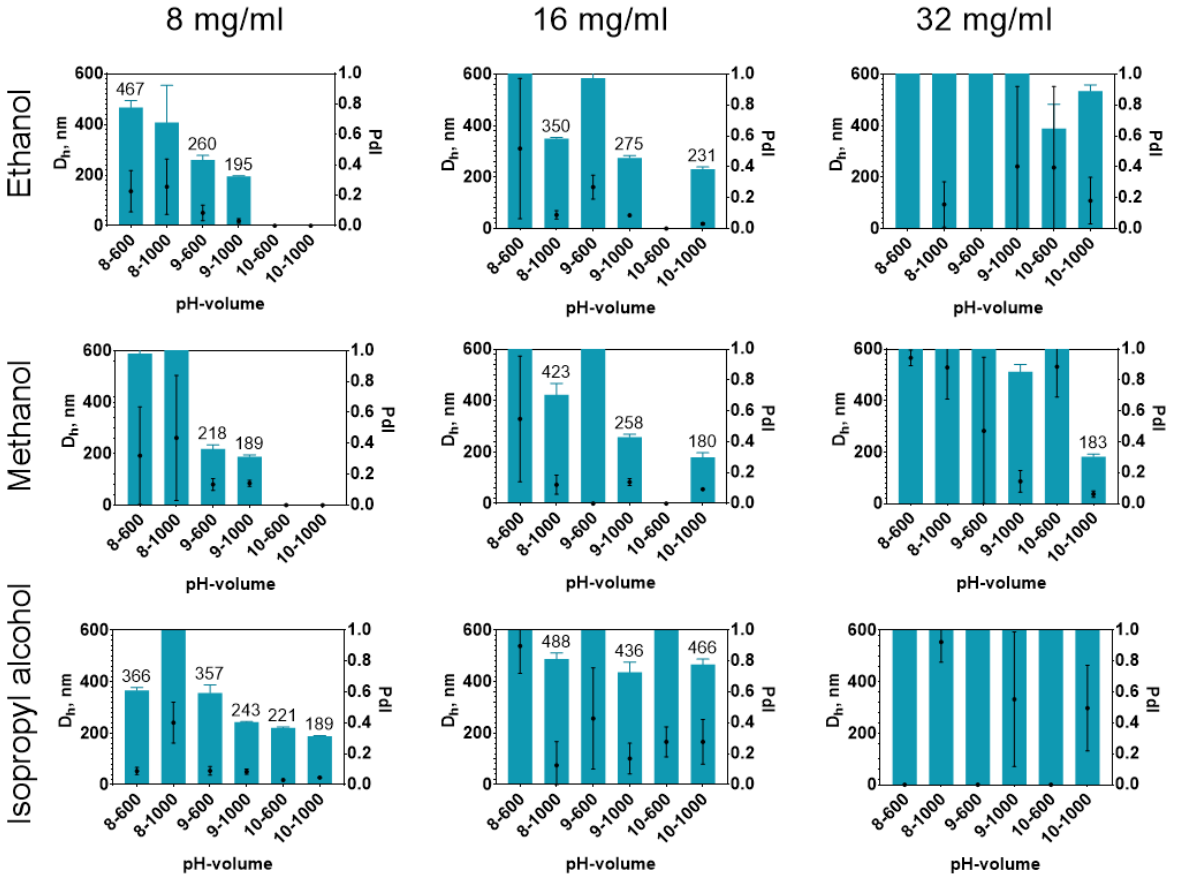

Figure S1. Dependence of the size and polydispersity of gelatin nanoparticles on pH, initial gelatin concentration, and type and volume of desolvating agent. Preliminary experiment, desolvation of 200 µLgelatin B, 75 bloom solution in centrifuge tubes. No bar means that nanoparticles were nor formed. Initial gelatin concentrations are given at the top of the figure. D_h - hydrodynamic diameter, PdI - polydispersity index. Mean values of three batches are shown, mean ± SD.

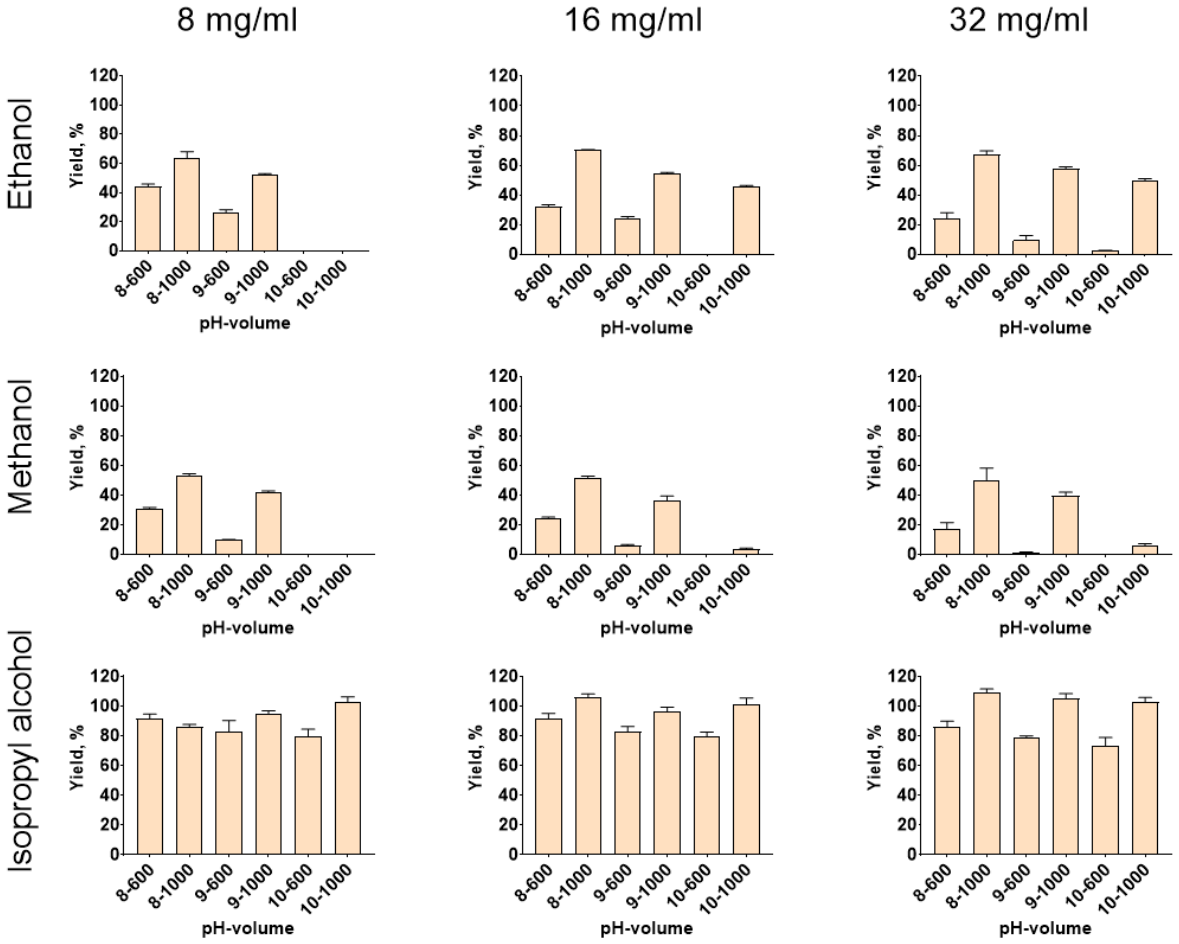

Figure S2. Dependence of the yield of gelatin nanoparticles on pH, initial gelatin concentration, and type and volume of desolvating agent. Preliminary experiment, desolvation of 200 µL gelatin B, 75 bloom solution in centrifuge tubes. No bar means that nanoparticles were nor formed. Initial gelatin concentrations are given at the top of the figure. Mean values of three batches are shown, mean ± SD.

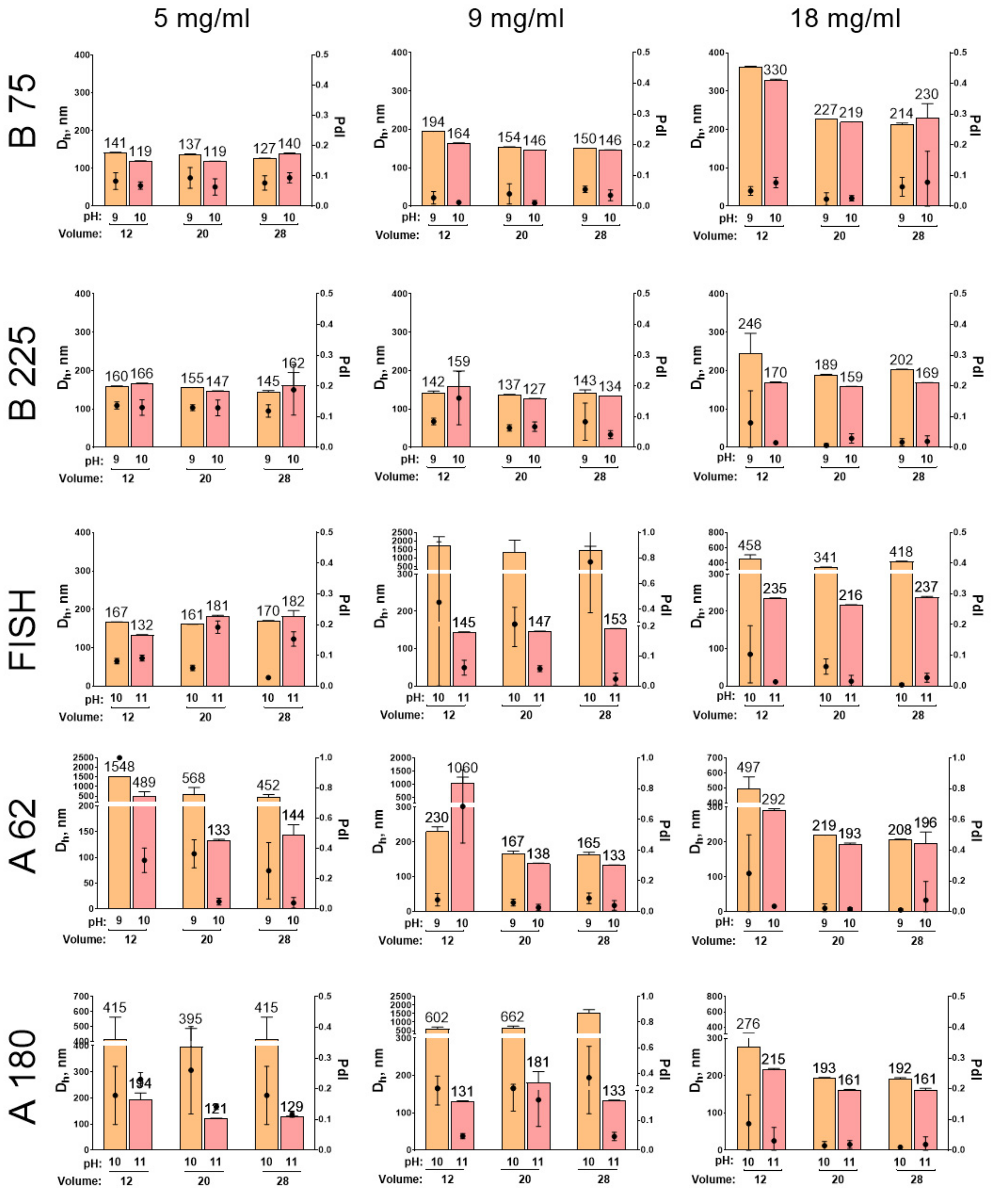

Figure S3. Size of gelatin nanoparticles prepared at different conditions using isopropyl alcohol as a poor solvent. Initial gelatin concentrations are given at the top of the figure. D_h - hydrodynamic diameter, PdI - polydispersity index. Mean values of three technical replicates are shown, mean ± SD.

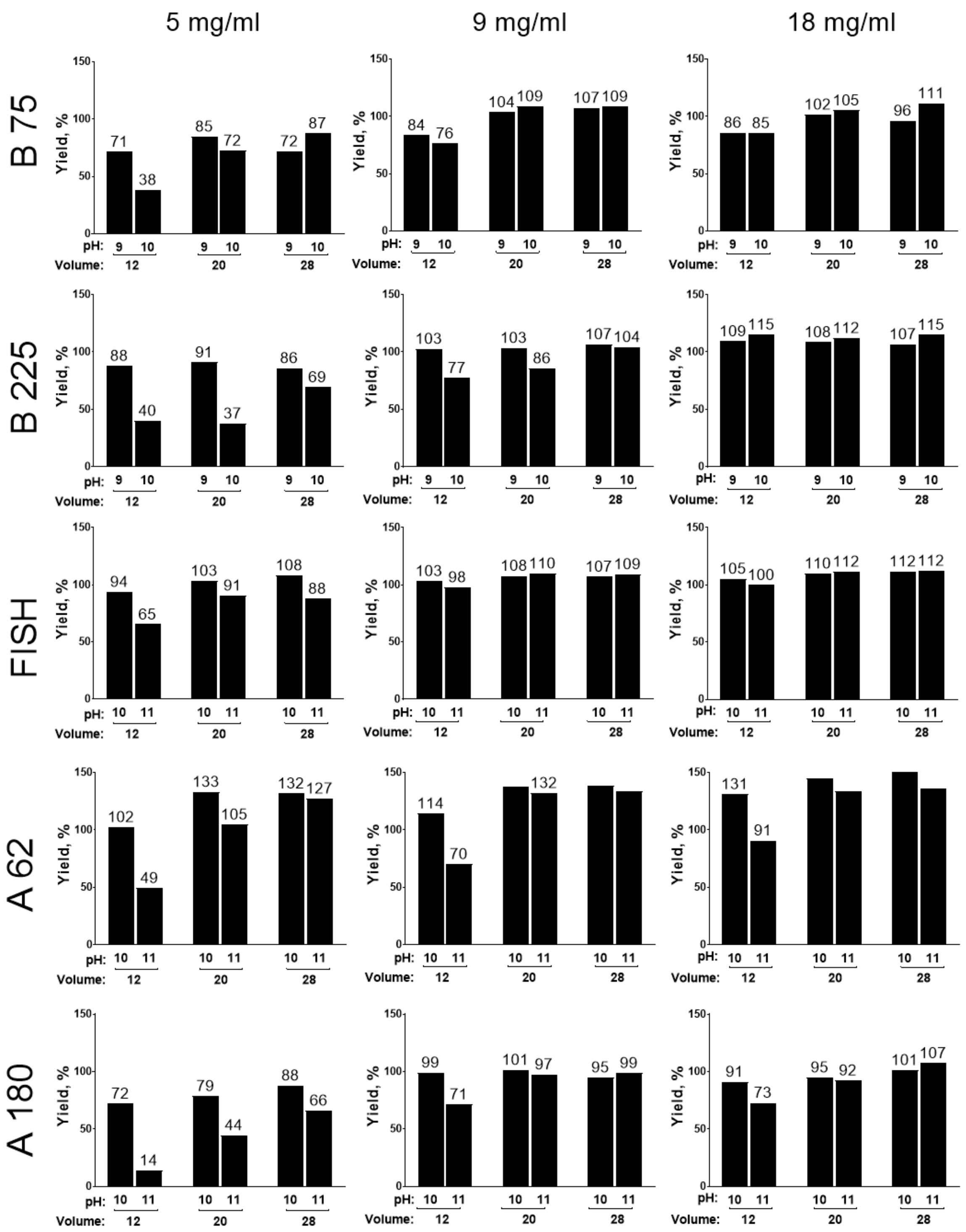

Figure S4. Yield of gelatin nanoparticles prepared at different conditions using isopropyl alcohol as a poor solvent. Initial gelatin concentrations are given at the top of the figure.

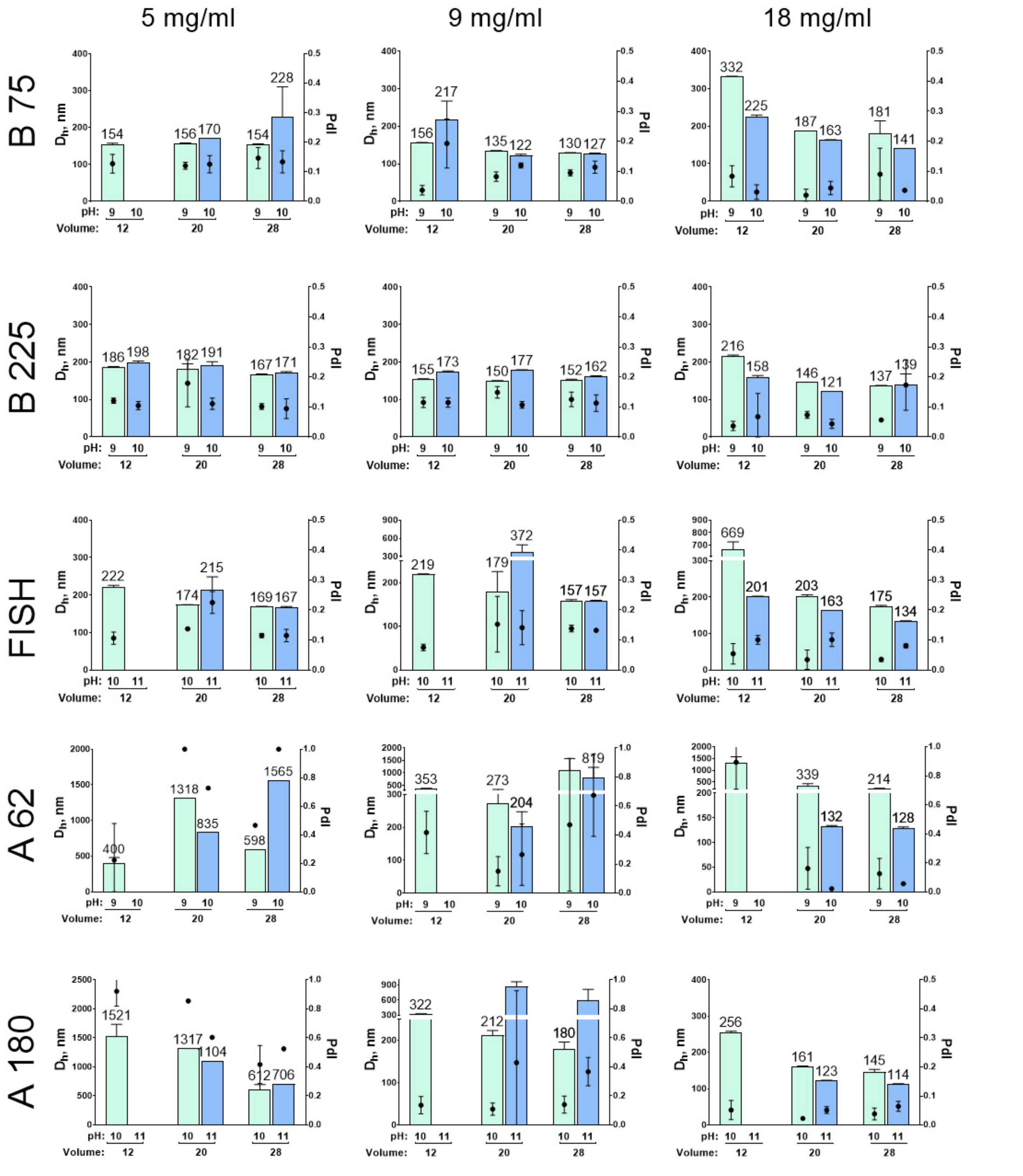

Figure S5. Size of gelatin nanoparticles prepared at different conditions using ethanol as a poor solvent. Initial gelatin concentrations are given at the top of the figure. No bar means that nanoparticles were nor formed. D_h - hydrodynamic diameter, PdI - polydispersity index. Mean values of three technical replicates are shown, mean ± SD.

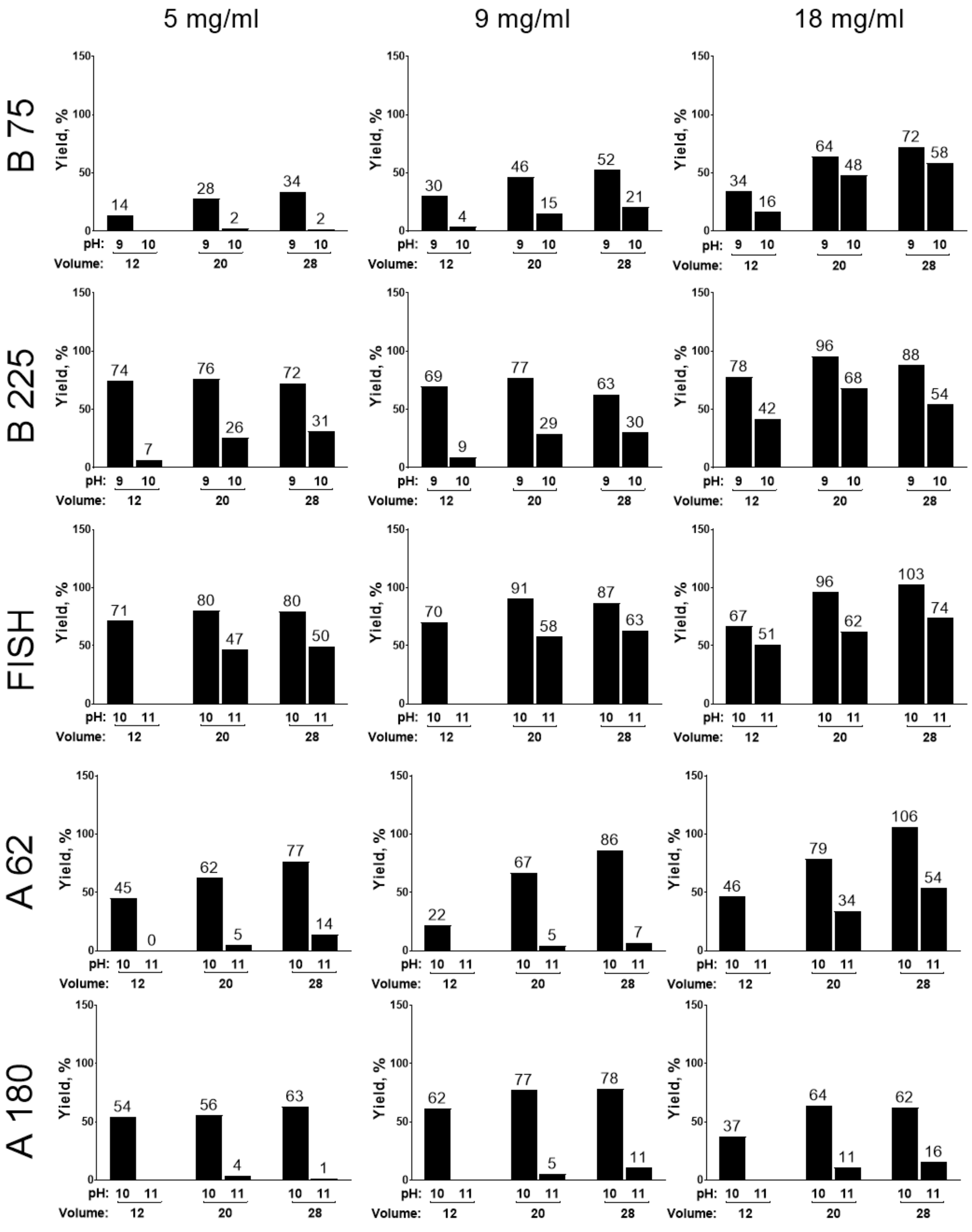

Figure S6. Yield of gelatin nanoparticles prepared at different conditions using ethanol as a poor solvent. Initial gelatin concentrations are given at the top of the figure. No bar means that nanoparticles were not formed.

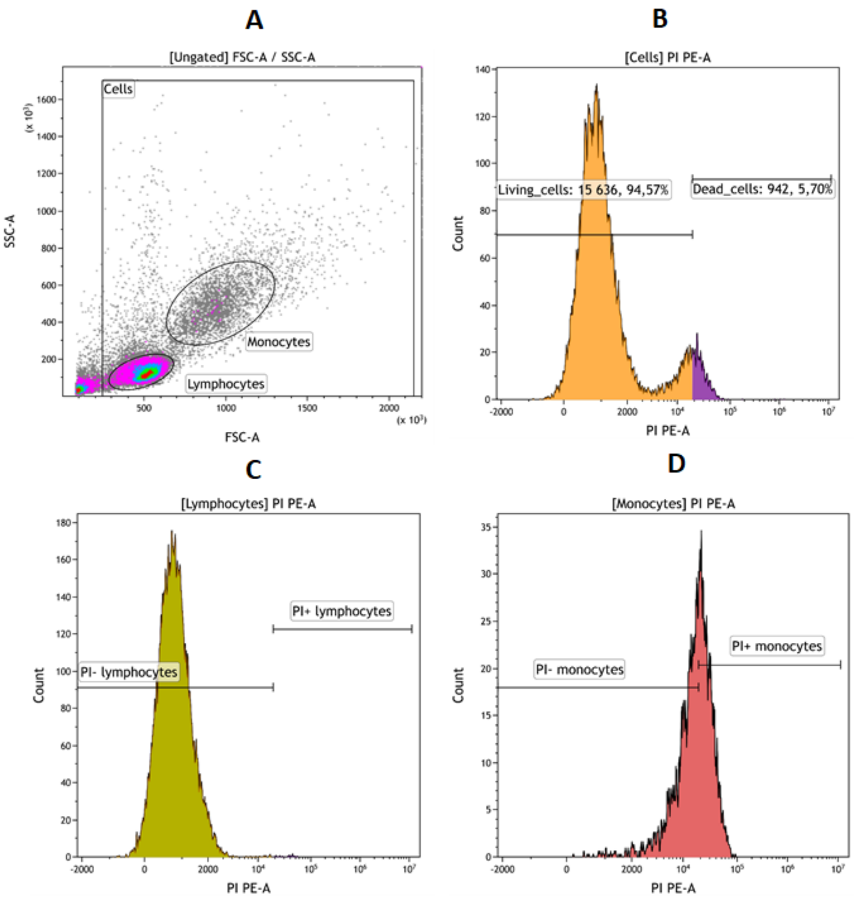

Figure S7. Fluorescence of monocytes engulfing particles in the emission spectrum of propidium iodide in an unstained sample with particles at a concentration of 1000 µg/mL. (**A**) - gating of PBMC (cells), lymphocytes, and monocytes on the light scatter dot plot; (**B**) - all PBMC are displayed on the histogram; (**C**) - the histogram the of lymphocytes gate; (**D**) - the histogram shows the gate of monocytes (some of the cells get into the gate of dead cells, set by unstained control without particles).

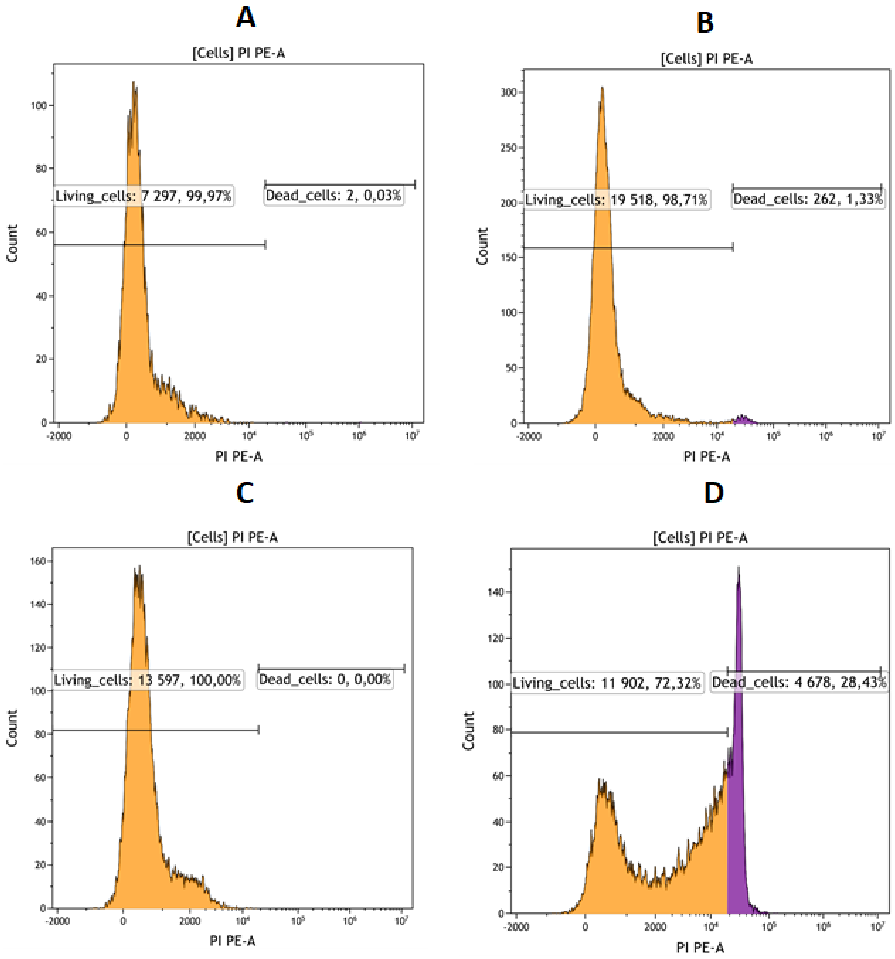

Figure S8. Gating of living and dead cells on unstained and stained controls without particles (**A**) - unstained control with WFI, (**B**) - stained control with WFI, (**C**) - unstained control with DMSO, (**D**) - stained control with DMSO). WFI - water for injections.

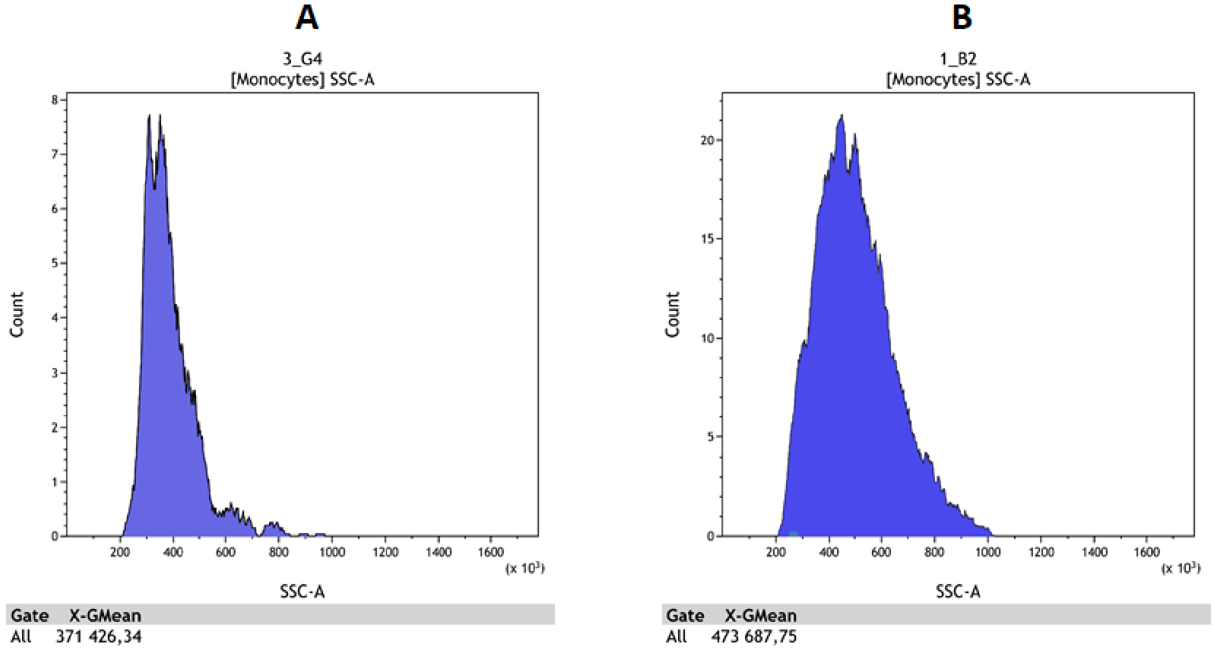

Figure S9. SSC histograms of monocyte gate in negative control without particles with water (**A**) and in the sample with 1000 µg/mL of particles (**B**). The geometric means of SSC intensities are indicated below the histograms.

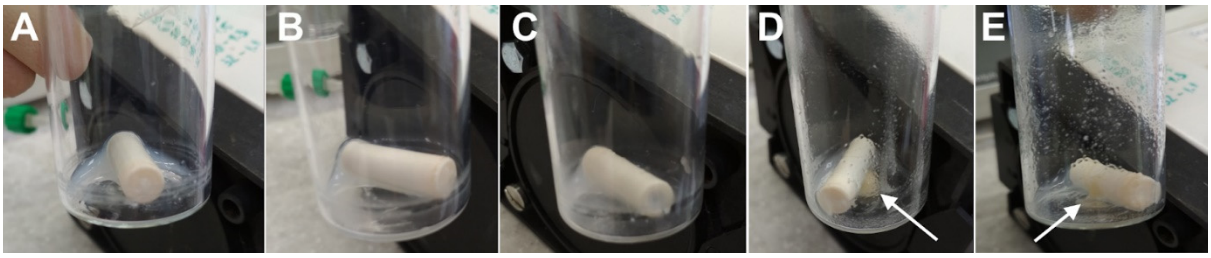

Figure S10. Bottom of the glass vials and magnets after synthesis of gelatin nanoparticles under stirring from the mixtures of gelatins (**A**) with bloom values of 300 and 62. A - A300:A62=4:0; (**B**) - A300:A62=3:1; (**C**) - A300:A62=2:2; (**D**) - A300:A62=1:3; (**E**) - A300:A62=0:4. Large gelatin aggregates are labeled with arrows.

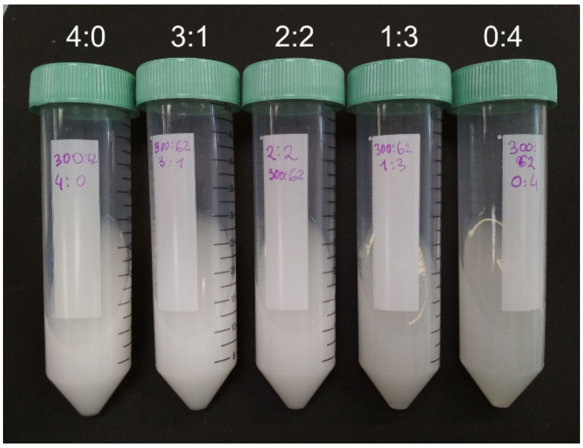

Figure S11. Gelatin nanoparticles prepared under stirring from the mixtures of gelatins A with bloom values of 300 and 62 (see text for details). Corresponding A300:A62 volume ratios are specified.

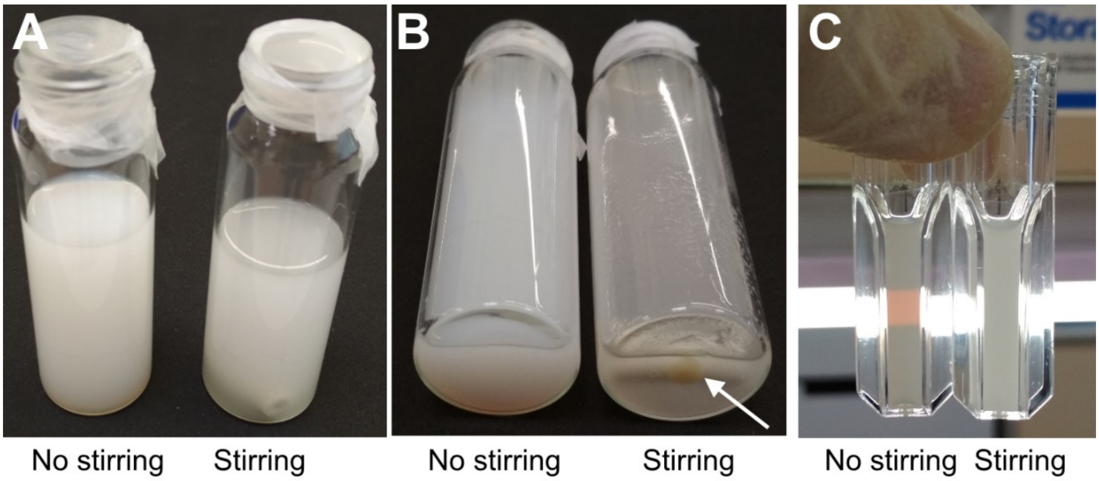

Figure S12. Gelatin nanoparticles prepared from gelatin A, 62 bloom by one-time addition of isopropyl alcohol without stirring and by dropwise addition of isopropyl alcohol under stirring. Large gelatin aggregates are labeled with arrows. (**A**, **B)** – vials with nanoparticles. (**C)** – visual assessment of thenanoparticle suspension turbidity

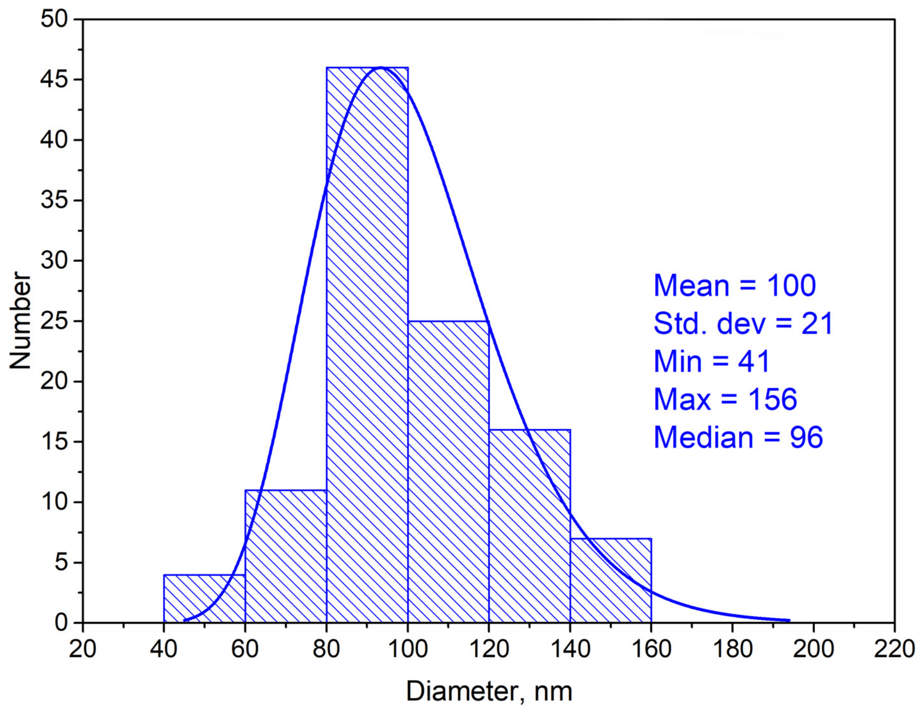

Figure S13. Size distribution of B75-1 nanoparticles obtained by TEM.

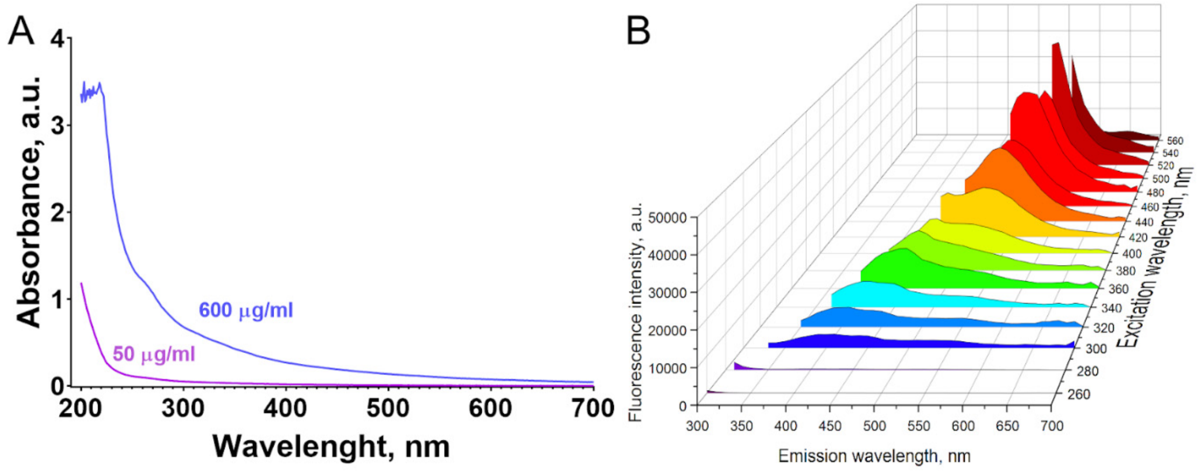

Figure S14. (A) - absorbance spectrum of B75-1 nanoparticles in water. The final concentration of nanoparticles is 50 and 600 μg/mL. (**B)** - emission spectra of B75-1 nanoparticles at various excitation wavelengths.

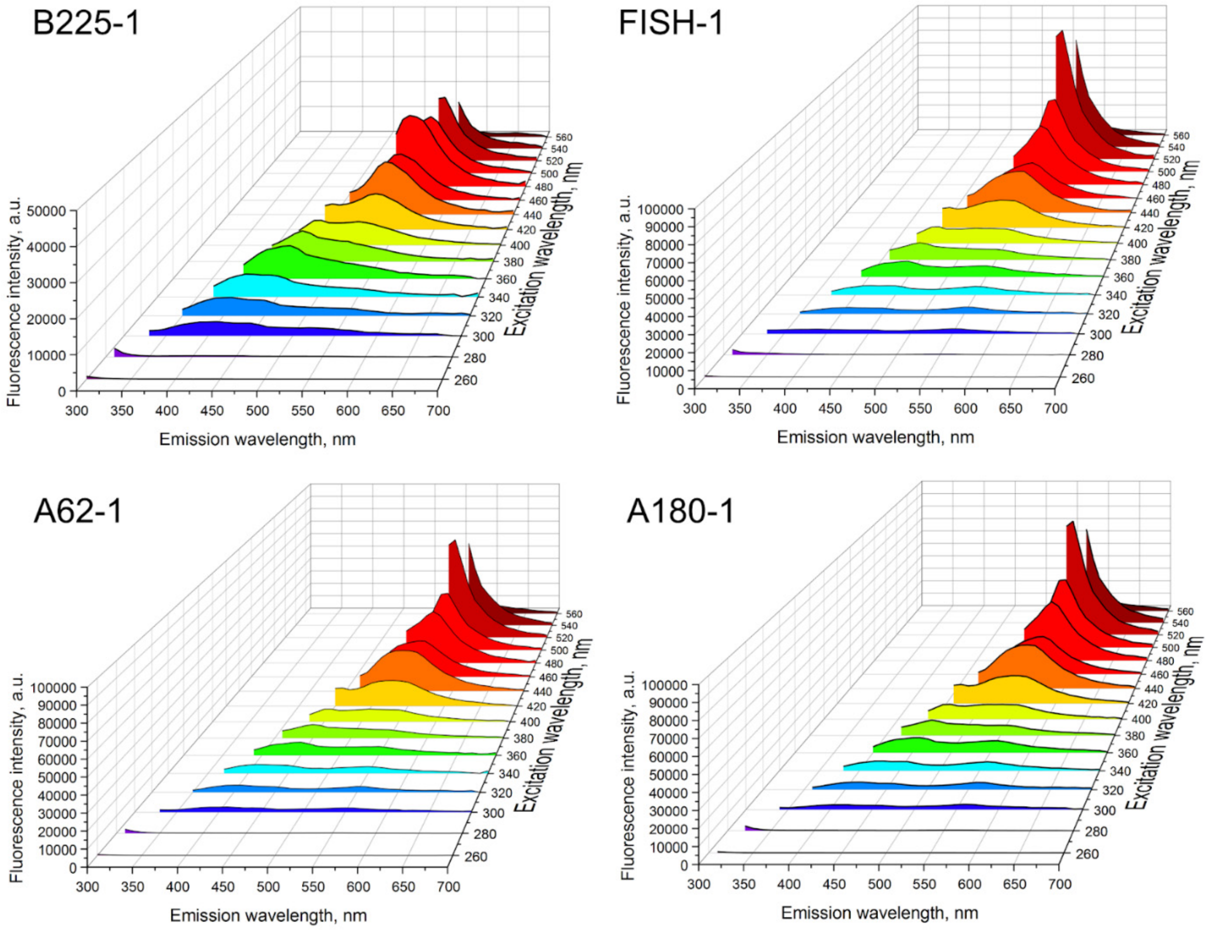

Figure S15. Emission spectra of gelatin nanoparticles at various excitation wavelengths.

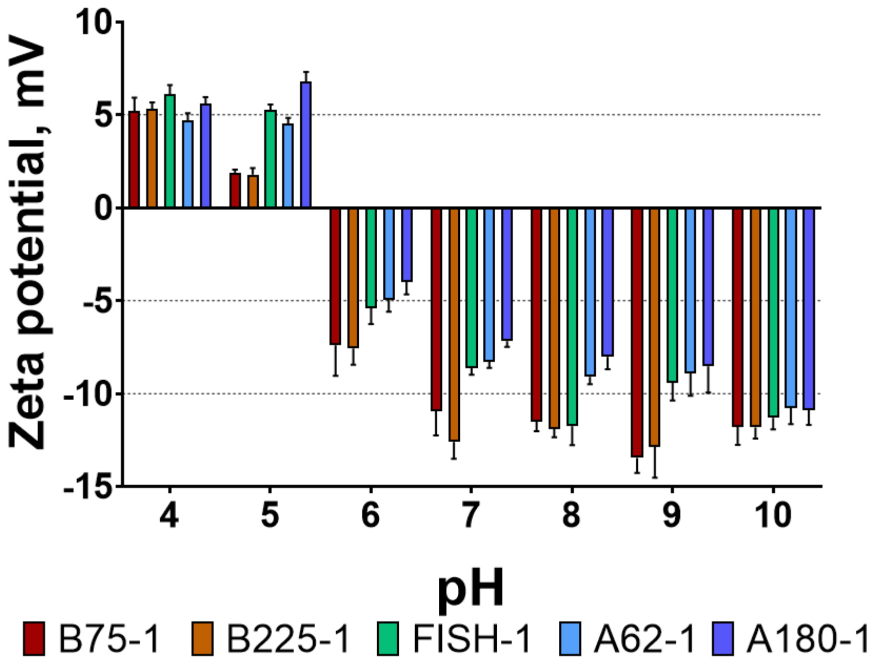

Figure S16. Zeta potential of gelatin nanoparticles at the different pH values. Mean values of three technical replicates are shown, mean ± SD.

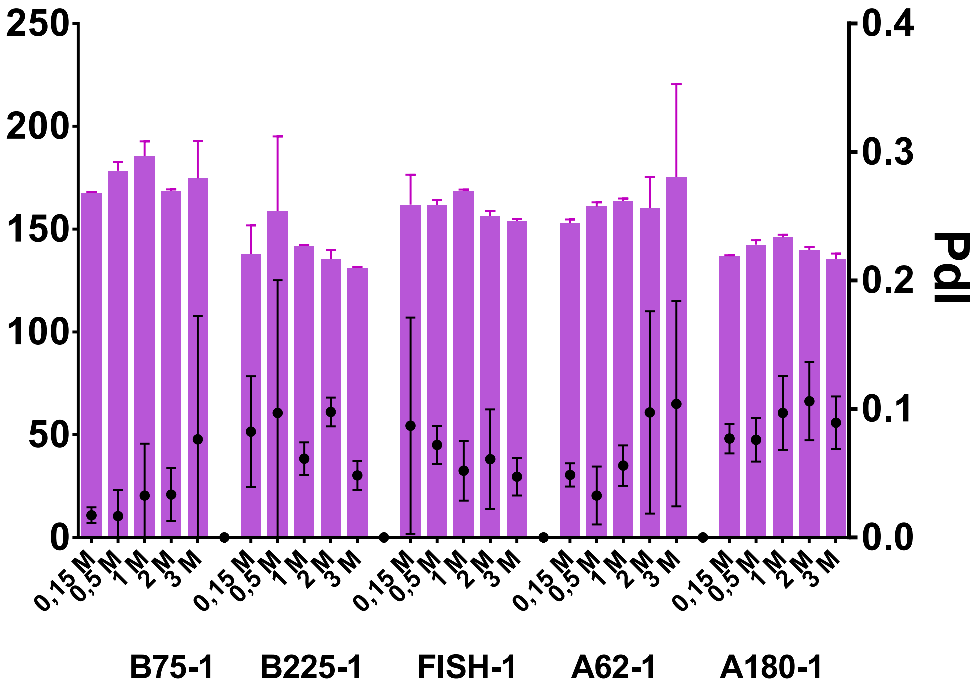

Figure S17. Colloidal stability of gelatin nanoparticles at different salt concentrations. D_h - hydrodynamic diameter, PdI - polydispersity index. Mean values of three technical replicates are shown, mean ± SD.

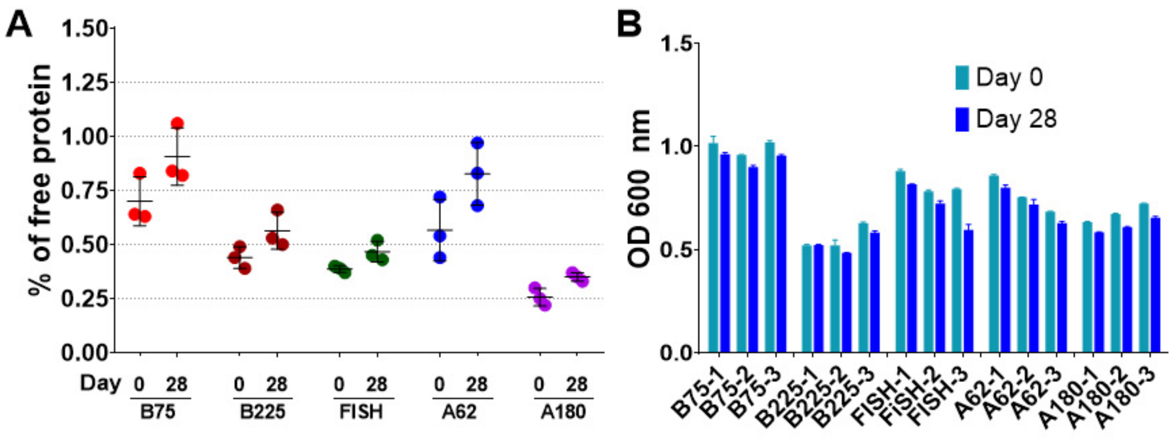

Figure S18. Storage stability of gelatin nanoparticles: (**A**) - the percentage of free protein in relation to nanoparticle concentration; (**B**) - turbidity of gelatin nanoparticle suspensions at days 0 and 28. Mean values of three technical replicates are shown, mean ± SD.

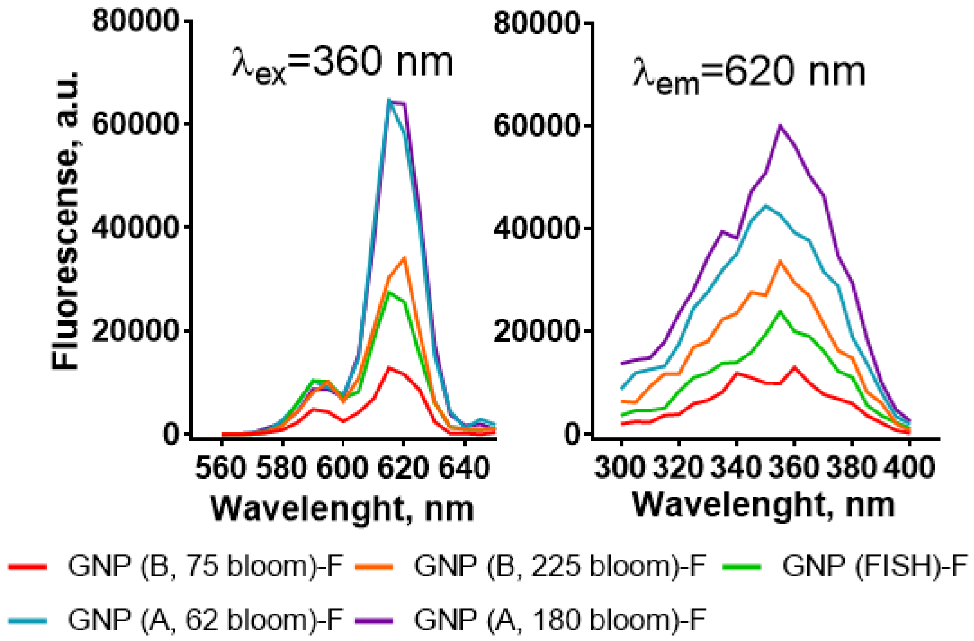

Figure S19. Emission and excitation spectra of gelatin nanoparticles loaded with europium complexes. Nanoparticles prepared from gelatin B, 75 bloom were diluted 1:1000, other nanoparticles were diluted 1:100.

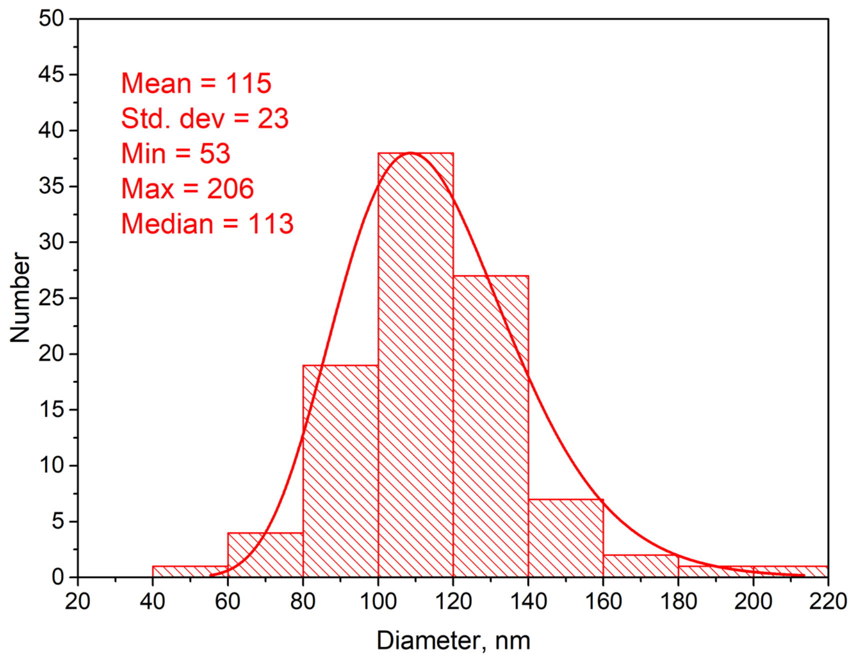

Figure S20. Size distribution of europium loaded gelatin nanoparticles (gelatin B, 75 bloom) obtained by TEM.

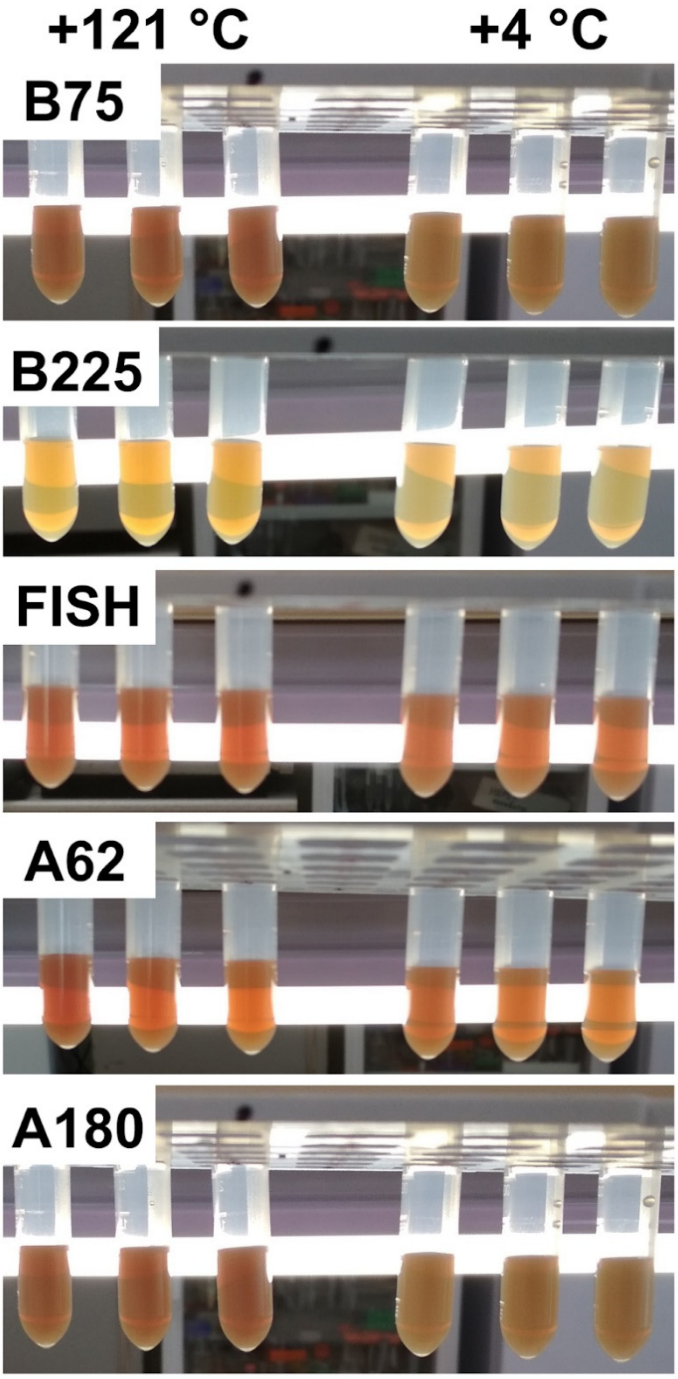

Figure S21. Color of autoclaved and non-autoclaved gelatin nanoparticle suspensions.

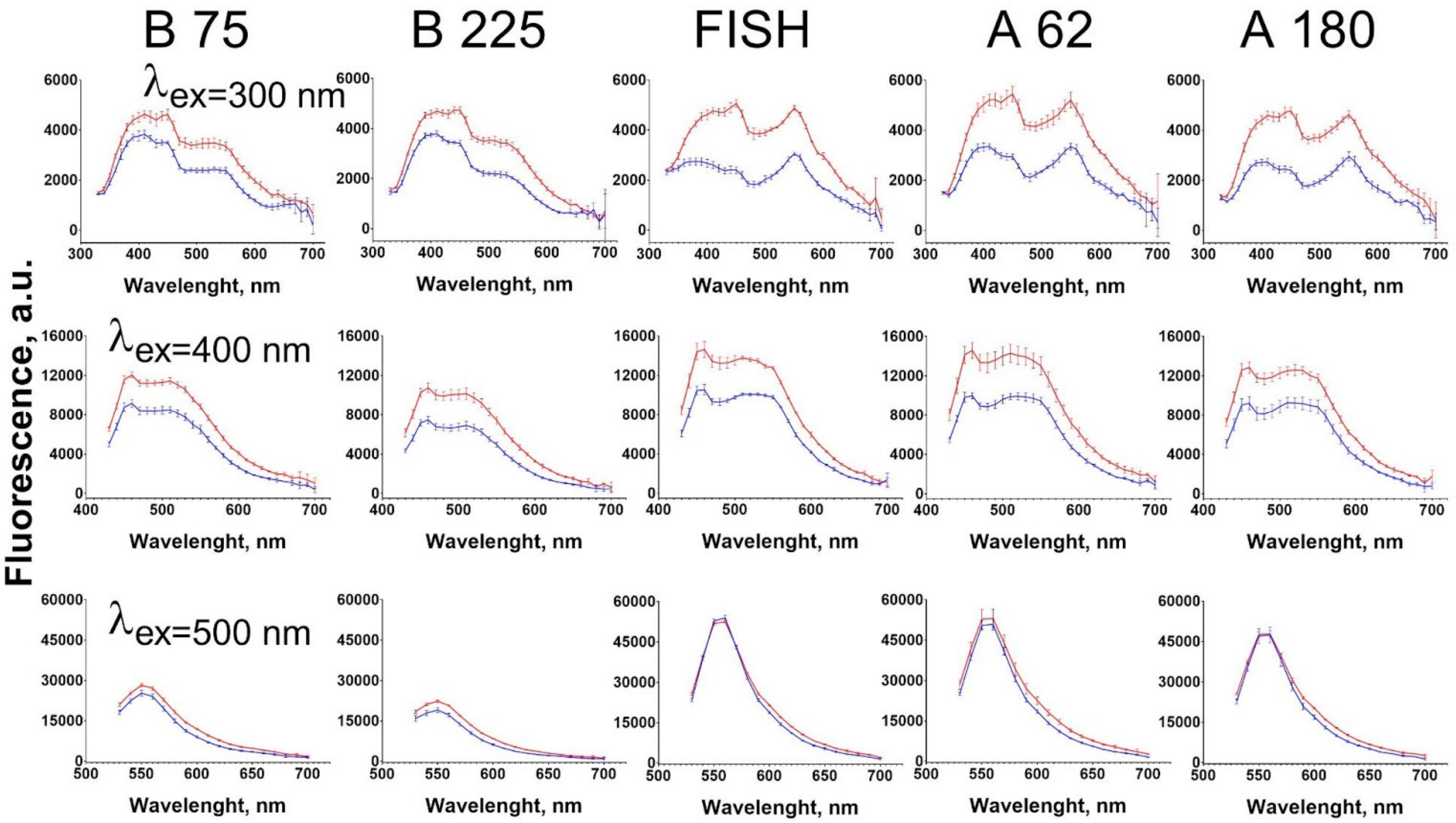

Figure S22. Emission spectra of non-autoclaved (blue) and autoclaved (red) gelatin nanoparticles at excitation wavelengths of 300 (top row), 400 (middle row), and 500 nm (bottom row). Mean fluorescence intensity values of three batches are shown, mean ± SD.

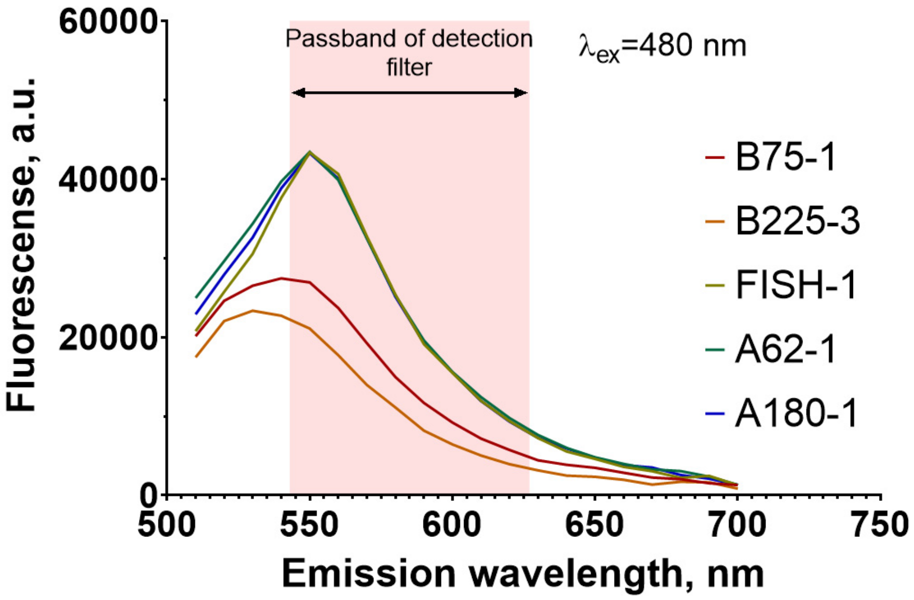

Figure S23. Fluorescence intensity of gelatin nanoparticles in the passband of detection filter.

Table S1. Yields of gelatin nanoparticles prepared by various methods.

No.	Method	Yield	Gelatin	Size	Reference
1	One-step desolvation	69–83% (standard batch size) 70% (scaled batch size)	High-bloom (300) gelatins A and B	150–300 nm	[90]
2	Two-step desolvation	1.5%	Gelatin type A 300 bloom	-	[90]
3	Nanoprecipitation	39–82%	Gelatin with bloom numbers from 75 to 300.	Paclitaxel-loaded nanoparticles 600–1000 nm	[91]
4	Nanoprecipitation	90 ± 5%	Gelatin type A 250 bloom	Doxorubicin-loaded gelatin nanoparticles, 100–200 nm	[92]
5	Two-step desolvation	70–75%	Gelatin type A 175 bloom	Antibody-labelled nanoparticles, 250–300 nm (PdI = 0.02)	[93]
6	Nanoprecipitation	73%	Gelatin type was not specified	191 nm	[94]
7	Nanoprecipitation	20–34%	Gelatin B 75 bloom	200–300 nm	[95]
8	Nanoprecipitation	23%	Gelatin B 75 bloom	200–300 nm	[96]
9	Two-step desolvation	less than 30%	Gelatin type A 175 bloom	140–200 nm	[97]
10	Two-step desolvation	26–51%	Gelatin type A 175 bloom	100–300 nm	[98]
11	One-step desolvation (high molecular weight fractions were desolvated)	up to 62%	Gelatin type A, high molecular weight fractions	50–260 nm	[99]

References

85. Weber, C., Coester, C., Kreuter, J., Langer, K. Desolvation process and surface characterisation of protein nanoparticles. *Int. J. Pharm*. **2000**, *194*, 91–102. DOI: 10.1016/s0378-5173(99)00370-1

86. Azarmi, S., Huang, Y., Chen, H., McQuarrie, S., Abrams, D., Roa, W., Finlay, W.H., Miller, G.G., & Löbenberg, R. Optimization of a two-step desolvation method for preparing gelatin nanoparticles and cell uptake studies in 143B osteosarcoma cancer cells. *J. Pharm. Pharm. Sci.* **2006**, *9*,124–132.

87. Ofokansi, K., Winter, G., Fricker, G., Coester, C. Matrix-loaded biodegradable gelatin nanoparticles as new approach to improve drug loading and delivery. *Eur J Pharm Biopharm*. **2010**, *76*, 1–9. DOI: 10.1016/j.ejpb.2010.04.008

88. Yoshikawa, H., Hirano, A., Arakawa, T., Shiraki, K. Effects of alcohol on the solubility and structure of native and disulfide-modified bovine serum albumin. *Int. J. Biol. Macromol.* **2012**, *50*, 1286–1291. DOI: 10.1016/j.ijbiomac.2012.03.014

89. Tyllianakis, P.E., Kakabakos, S.E., Evangelatos, G.P., Ithakissios, D.S. Direct colorimetric determination of solid-supported functional groups and ligands using bicinchoninic acid. *Anal. Biochem*. **1994**, *219*, 335–340. DOI: 10.1006/abio.1994.1273
90. Geh, K.J., Hubert, M., Winter, G. Optimisation of one-step desolvation and scale-up of gelatine nanoparticle production. *J. Microencapsulation*, **2016**, *33*, 595–604. DOI: 10.1080/02652048.2016.1228706
91. Lu, Z., Yeh, T.-K., Tsai, M., Au, J.L.-S., Wientjes, M.G. Paclitaxel-loaded gelatin nanoparticles for intravesical bladder cancer therapy. *Clin. Cancer Res.* **2004**, *10*, 7677–7684. DOI: 10.1158/1078-0432.CCR-04-1443
92. Leo, E., Vandelli, M.A., Cameroni, R., Forni, F. Doxorubicin-loaded gelatin nanoparticles stabilized by glutaraldehyde: Involvement of the drug in the cross-linking process. *Int J Pharm.* **1997**, *155*, 75–82. DOI: 10.1016/S0378-5173(97)00149-X
93. Balthasar, S. Charakterisierung proteinbasierter Nanopartikel zum Transport von Oligonukleotiden für eine Rezeptor-vermittelte Zellaufnahme. Dissertation zur Erlangung des Doktorgrades der Naturwissenschaften. 2005. Available online: http://d-nb.info/978388593/34 (accessed on 15 July 2021).
94. Das R.P., Chakravarti S., Patel S.S., Lakhamje P., Gurjar M., Gota V., Singh B.G., Kunwar A. Tuning the pharmacokinetics and efficacy of irinotecan (IRI) loaded gelatin nanoparticles through folate conjugation. *Int J Pharm.* **2020** *586*, 119522. DOI: 10.1016/j.ijpharm.2020.119522.
95. Khan, S.A., Ali, H., Ihsan, A., Sabir, N. Tuning the size of gelatin nanoparticles produced by nanoprecipitation. *Colloid J.* **2015**, *77*, 672–676. DOI: 10.1134/s1061933x15050105
96. Lee, E.J., Khan, S.A., Park, J.K., Lim, K.-H. Studies on the characteristics of drug-loaded gelatin nanoparticles prepared by nanoprecipitation. *Bioprocess Biosyst. Eng.* **2011**, *35*, 297–307. DOI: 10.1007/s00449-011-0591-2
97. Fuchs, S. Gelatin Nanoparticles as a modern platform for drug delivery: formulation development and immunotherapeutic strategies. Dissertation. Ludwig Maximilian University of Munich. Munich. 29. July 2010.
98. Zwiorek, K. Gelatin Nanoparticles as Delivery System for Nucleotide-Based Drugs. Dissertation. Ludwig Maximilian University of Munich. Munich. 3 August 2006
99. Shamarekh, K.S., Gad, H.A., Soliman, M.E., Sammour, O.A. Towards the production of monodisperse gelatin nanoparticles by modified one step desolvation technique. *J. Pharm. Invest.* **2020**, *50*, 189–200. DOI: 10.1007/s40005-019-00455-x
